# Characterization of DNA Repair Deficient Strains of *Chlamydomonas reinhardtii* Generated by Insertional Mutagenesis

**DOI:** 10.1371/journal.pone.0105482

**Published:** 2014-08-21

**Authors:** Andrea Plecenikova, Miroslava Slaninova, Karel Riha

**Affiliations:** 1 Gregor Mendel Institute, Austrian Academy of Sciences, Vienna Biocenter (VBC), Vienna, Austria; 2 Department of Genetics, Faculty of Natural Sciences, Comenius University in Bratislava, Bratislava, Slovakia; 3 Central European Institute of Technology (CEITEC), Masaryk University, Brno, Czech Republic; Louisiana State University and A & M College, United States of America

## Abstract

While the mechanisms governing DNA damage response and repair are fundamentally conserved, cross-kingdom comparisons indicate that they differ in many aspects due to differences in life-styles and developmental strategies. In photosynthetic organisms these differences have not been fully explored because gene-discovery approaches are mainly based on homology searches with known DDR/DNA repair proteins. Here we performed a forward genetic screen in the green algae *Chlamydomonas reinhardtii* to identify genes deficient in DDR/DNA repair. We isolated five insertional mutants that were sensitive to various genotoxic insults and two of them exhibited altered efficiency of transgene integration. To identify genomic regions disrupted in these mutants, we established a novel adaptor-ligation strategy for the efficient recovery of the insertion flanking sites. Four mutants harbored deletions that involved known DNA repair factors, DNA Pol zeta, DNA Pol theta, SAE2/COM1, and two neighbouring genes encoding ERCC1 and RAD17. Deletion in the last mutant spanned two *Chlamydomonas*-specific genes with unknown function, demonstrating the utility of this approach for discovering novel factors involved in genome maintenance.

## Introduction

DNA double strand breaks (DSBs) pose a serious threat to genome integrity as their erroneous repair may lead to chromosomal rearrangements with potentially lethal consequences for an organism. The response to DSBs elicits a highly organized and complex cellular program, called the DNA damage response (DDR), which sets in motion processes that mitigate the adverse effects of DNA damage and facilitate DNA repair. Broken DNA is usually repaired by one of two mechanistically distinct pathways: homologous recombination (HR) and non-homologous end joining (NHEJ). While HR uses a homologous DNA strand as a template for error-free repair, NHEJ is inherently error-prone and does not rely on sequence homology. The preferred mode of repair and cellular consequences of DDR varies between organisms and is also dependant on cell type and cell cycle context [1], [2]. For example, while HR is the preferred mode of repair in many unicellular organisms such as budding and fission yeast, NHEJ is the prevalent pathway in plants and animals.

In many aspects, plants seem to respond differently to DNA insults than do animals. The constant risk of tumor formation in animals has led to evolution of DDR that assures precise genome maintenance, often resulting in apoptotic death of damaged cells. The lack of such a strong selective constraint presumably permitted evolution of a more relaxed DDR in plants, making them more tolerant to genome damage [3], [4]. Furthermore, plant cells are exposed to high levels of genotoxic stress resulting from long-term exposure to solar ultraviolet (UV) irradiation, photosynthesis and extended periods of desiccation [5], [6]. Thus, some features of plant DDR and DSB repair may deviate from models primarily established from studies in yeasts and mammals. Functional characterization of plant DDR and DSB repair is mainly limited to a few model organisms including *Arabidopsis thaliana*, rice, maize, and the moss *Physcomytrella patens* [6], [7], [8]. The key experimental strategy for DDR gene discovery in plants is based on homology searches for conserved DNA repair proteins known from yeast and animals [9], [10]. Forward genetic screens have a much higher potential to identify unknown genes with novel DDR and DSB repair functions, and they have been successfully applied for dissecting the mechanism of meiotic recombination [11], [12]. However, only a few screens were performed with the aim to identify novel components of somatic DSB repair [13], [14], [15]. This is partially due to the complicated nature of these screens, as the scored phenotype is often represented by sensitivity to a genotoxic treatment and hence the death of the plant that carries the desired mutation.

In this study we exploited the potential of the unicellular green algae *Chlamydomonas reinhardtii* in discovery of genes related to DDR and DSB repair in the plant kingdom. *C. reinhardtii* is an established model that is traditionally used for studying photosynthesis and cell motility. Interest in this organism has also been sparked by prospects of using algae as source of hydrogen or lipids for biofuel production. In addition, sharing a common ancestor 1.1 billion years ago, *C. reinhardtii* provides an excellent complementary model to higher plants for comparative gene analyses within the plant kingdom [16]. Whereas unicellular *C. reinhardtii* appears to be a relatively simple organism, in regards to genome complexity and mechanisms that govern genome maintenance and expression it is comparable with higher eukaryotes. The draft genome sequence revealed more than 15,000 genes that contain a large number of introns and numerous repetitive DNA sequences and transposable elements [17]. The presence of DNA methylation, siRNA and microRNAs indicates that epigenetic and post-transcriptional regulation of gene expression is more complex than in many other unicellular models [18], [19]. In addition, *C. reinhardtii* displays an extremely low efficiency of HR, which is unusual for a unicellular organism [20] and similar to higher plants. *C. reinhardtii* therefore offers the unique opportunity to study some of the cellular processes that are featured in higher eukaryotes while providing the advantages of a unicellular experimental system.

Here, we performed a forward genetic screen of a population of *C. reinhardtii* mutagenized by insertional mutagenesis for sensitivity to genotoxic stress and describe an efficient strategy for identification of disrupted loci. We characterized five mutants and determined that they encompass deletions in known DNA repair genes, which validated our approach, as well as in genes with unknown functions, demonstrating the potential of this approach in identification of novel DDR related genes.

## Materials and Methods

### Strain and growth media

The *C. reinhardtii* strain *cw15-302 arg2* [21] was obtained from the laboratory of Dr. Christoph Beck (University of Freiburg, Germany). Cells were grown under constant light at 22°C in Tris-acetate phosphate media (TAP) supplemented with L-arginine (100 mg/L) and with 1% agar when solid medium was required [22].

### Genetic screen

**Figure 1:**
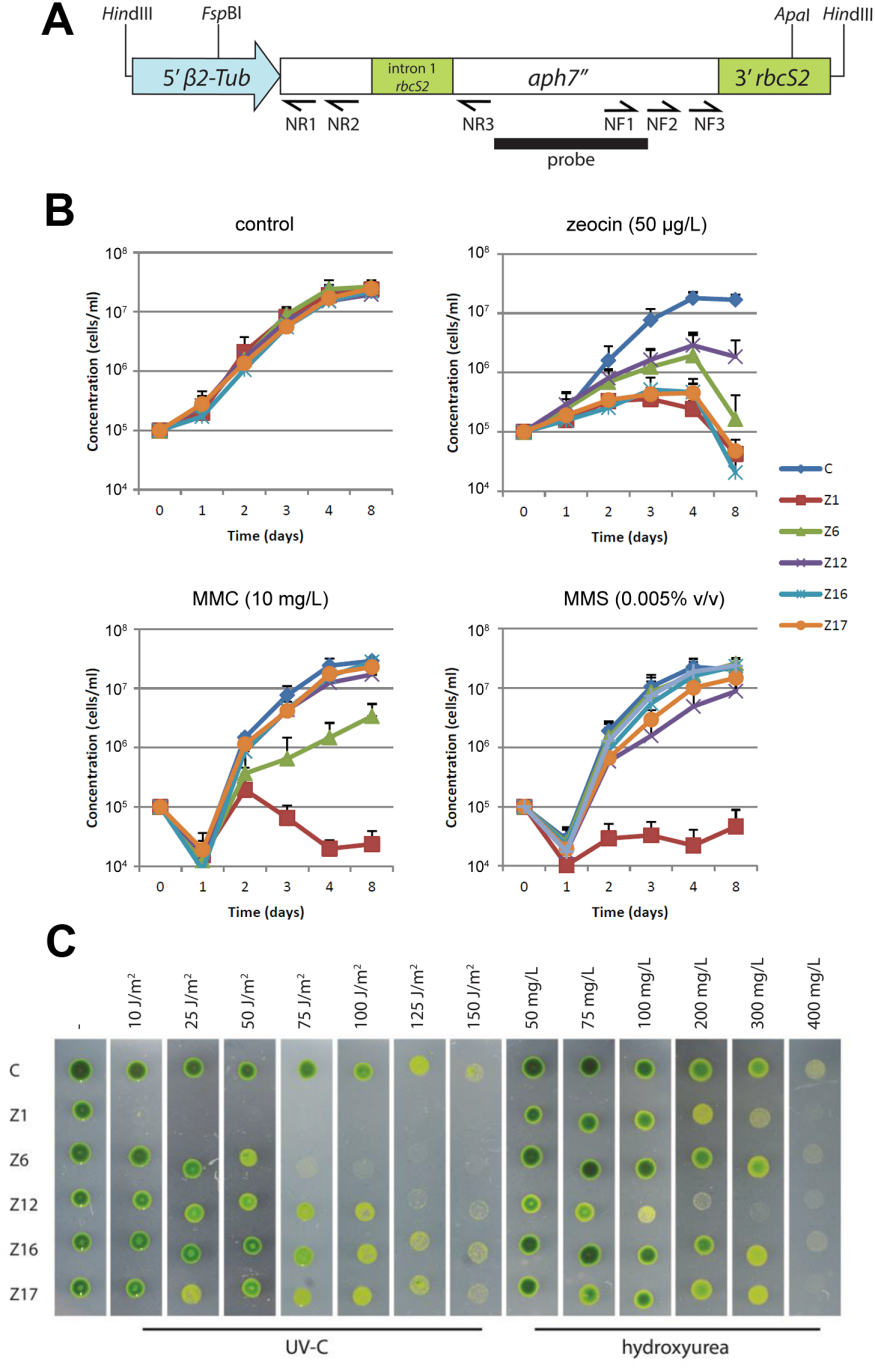
Sensitivity of *C. reinhardtii* mutant strains to genotoxic treatments. (A) Structure of the *aph7"* construct used for the insertional mutagenesis. Primers (arrows), restriction sites and the region used as a probe for Southern hybridization are indicated. (B) Growth curves of mutant and the parental *cw15-302 arg2* strains (denoted as C) in TAP liquid media supplemented with zeocin, MMC or MMS. (C) Growth of mutant strains on TAP plates exposed to increasing dose of UV-C or HU. Pictures were taken five days after inoculation.

Plasmid pHyg3 [23] was digested with *Hin*dIII and the linear fragment containing the chimeric *aph7*″ selection marker gene was used as the insertional cassette (Figure 1A). Insertional mutagenesis of *C. reinhardtii* cells was performed by the glass-bead transformation method [24]. Immediately after transformation, cells were spread onto selective TAP plates containing 10 mg/L of hygromycin B (Calbiochem) and grown for 1 week. Transformed colonies resistant to hygromycin B were resuspended in sterile distilled water and replicated to one set of replica TAP plates with or without 300 µg/L of zeocin (Duchefa Biochemie) and to the second set of replica plates, one of which was irradiated with UV-C (70 J/m^2^) immediately after plating (Stratalinker UV Crosslinker 2400, Stratagene). For the UV-C sensitivity screen, the plates were incubated for one day in the dark to avoid photoreactivation. Colonies sensitive to the selection conditions were maintained on TAP plates and in the subsequent 3–6 months re-screened at least twice for the sensitivity to zeocin and UV-C irradiation.

### Genotoxicity tests

Sensitivity to hydroxyurea (HU) was assessed by inoculating cells on TAP plates supplemented with 50–400 mg/L HU (Sigma-Aldrich). Sensitivity to UV-C irradiation was examined by scoring cell growth on TAP media exposed to 10 J/m^2^–150 J/m^2^ UV-C (Stratalinker UV Crosslinker 2400, Stratagene). Sensitivity to zeocin (50 µg/L), mitomycin C (MMC; 10 mg/L; Sigma-Aldrich) and methyl methanesulfonate (MMS; 0,005%) was tested by determining growth curves in TAP liquid media. Mutant and control strains were first grown for 3 days on TAP plates with L-arginine (100 mg/L) and hygromycin B (10 mg/L) under continuous light and then transferred to liquid TAP media supplemented with L-arginine and the tested drug. Cell density was determined in a Bürker chamber after 3 days (time point 0) and then again at the 24, 48, 72, 96 and 192 hours. Data from three independent experiments were collected and analyzed.

### Nucleic acid isolation and analysis

Total genomic DNA was isolated using a modified protocol according to [25]. 50 ml of *C. reinhardtii* culture grown for 3 days (∼2.10^7^ cells. ml^−1^) was pelleted by centrifugation, resuspended in 2×CTAB buffer [1.4 M NaCl, 0.1 M Tris ph 8.0, 0.02 M EDTA, 2% hexadecyltrimethylammoniumbromide (CTAB, Sigma)] and incubated for 1 h at 65°C. DNA was extracted with phenol:chloroform:isoamyl alcohol (25∶24∶1, v/v) and precipitated by isopropanol. RNA was removed by 30 min treatment with RNase (164 mg/L) at 37°C that was subsequently removed by phenol and chloroform:isoamyl alcohol (24∶1, v/v) extractions. DNA was purified by ethanol precipitation. Genomic DNA was subjected to Southern hybridization analysis according to standard protocols [26]. A PCR fragment amplified from the pHyg3 plasmid with primers HygR_PR1_F616 5′-GAGAGCACCAACCCCGTACTGG-3′ and HygR_PR1_R1196 5′-GTGAAGTCGACGATCCCGGT-3′ was radioactively labeled with [α ^32^P] dCTP and used as a probe.

### Isolation of insert-flanking regions by hairpin-PCR

2 µg of genomic DNA was digested with a restriction endonuclease according to supplier’s specifications and isopropanol precipitated. 1 µg of digested DNA was ligated to a hairpin adaptor using T4 DNA ligase (Thermo Fisher Scientific) for 16 h at laboratory temperature. The hairpin adaptor [27] compatible with the DNA ends generated by restriction digest was used in a final concentration of 1.6 µM. DNA was isopropanol precipitated and 40–80 ng were used as the template in PCR. Genomic DNA flanking the site of insertion was amplified by two rounds of PCR with the primer PETRA-B (5′-CTCTAGACTGTGAGACTTGGAGATG-3′) and nested insert specific primers complementary to the *aph7*″ gene (Figure 1A): NR1 (5′-CCAGTGCTCGCCGAACAGCT-3′), NR2 (5′-TCGTTCCGCAGGCTCGCGTA-3′), NF1 (5′-GAGACTCCCGCTACAGCCTG-3′) and NF2 (5′-CTGCACGACTTCGAGGTGTT CG-3′). PCR mixture consisted of 1×GoTaq Reaction Buffer (Promega), 0.2 mM dNTP, 0.5 µM PETRA-B, 0.5 µM NR1 or NF1, 40 ng–80 ng DNA, 3% DMSO, Go Taq DNA polymerase (Promega) and the reaction was performed with 35 cycles of 45 s at 94°C, 30 s at 64°C and 2 min 30 s at 72°C. 0.5 µl of PCR amplification product was used as the template for nested PCR with PETRA-B and NR2 or NF2 primers. PCR products were cloned into pGEM-T Easy Vector (Promega) or to pCR2.1-TOPO Vector (Invitrogen) and transformed to *E. coli* DH5α competent cells and sequenced using a standard procedure.

### Insert integration assay

*C. reinhardtii* strains were transformed by the glass-bead method with plasmids pUCARG7.8 and pUCBM20ΔARG [21] and transformation efficiency and HR/NHEJ ratios were calculated as previously described [28].

## Results

We generated a library of *C. reinhardtii* insertional mutants by transforming the cell-wall deficient strain *cw15-302 arg2* with the 1.7 kb fragment of the pHyg3 plasmid containing the chimeric *aph7*″ gene that confers resistance to hygromycin B [23] (Figure 1A). We used a glass-bead transformation technique and a low amount of DNA (500 ng) per transformation to decrease the chance of multiple insertions per genome [29]. In total, we generated 4588 transformants that were subsequently screened for growth on agar plates supplemented with zeocin (300 mg/L), an antibiotic that induces DSB, and for survival on plates exposed to UV-C irradiation (70 J/m^2^). After two rounds of screening we obtained 19 clones sensitive to zeocin, 52 clones sensitive to UV-C, and 9 clones sensitive to both treatments. However, only five mutant clones retained the phenotype after 3–6 months of subculturing. All five mutant strains, which we named Z1, Z6, Z12, Z16 and Z17, were originally selected for their inability to grow on agar plates supplemented with zeocin, and this phenotype was also confirmed by cultivation in liquid media (Figure 1B). Z1, Z16 and Z17 exhibited the highest growth retardation in the presence of zeocin, while Z6 showed intermediate sensitivity and the growth of Z12 mutants was least affected (Figure 1B and Table 1). The growth of clones Z1 and Z6 was also impaired by UV-C irradiation (Figure 1C, Table 1).

**Table 1:** Relative sensitivity to genotoxic treatments in respect to other mutants and the control strain *cw15-302 arg2* (+++ high sensitivity, ++ intermediate sensitivity, + mild sensitivity, −no sensitivity).

Mutant	Zeocin	UV	MMC	MMS	HU
Z1	+++	+++	+++	+++	+
Z6	++	++	+	−	−
Z12	+	+	−	++	+++
Z16	+++	−	−	−	−
Z17	+++	−	−	+	−

We next tested sensitivity of the mutants to the genotoxic drugs MMC, methyl MMS and HU. These drugs induce DNA damage by different mechanisms: MMC causes DNA interstrand cross-linking, MMS treatment methylates DNA which is believed to lead to replication fork stalling, and HU impairs production of deoxyribonucleotides, which inhibits DNA replication and results in stalled replication forks. MMC almost fully suppressed growth of the Z1 mutant, while proliferation of Z6 was impaired to lesser extent (Figure 1B, Table 1). Other strains were unaffected. Z1 also exhibited the highest sensitivity to MMS. A slight inhibition of growth by MMS was detected in Z12 and Z17 (Figure 1B, Table 1). HU had the strongest effect on Z12, exhibiting a discernable effect already at the lowest concentration of HU. A slight inhibition was also detected in the Z1 mutant (Figure 1C, Table 1). The observation that almost all mutants were sensitive to at least two independent genotoxic treatments validated the screening approach and suggested that the mutants we obtained are indeed impaired in DDR/DSB repair. Moreover, the differential sensitivity of the 5 mutant strains to particular treatments indicated a deficiency in different aspects of DDR and DNA repair.

**Figure 2:**
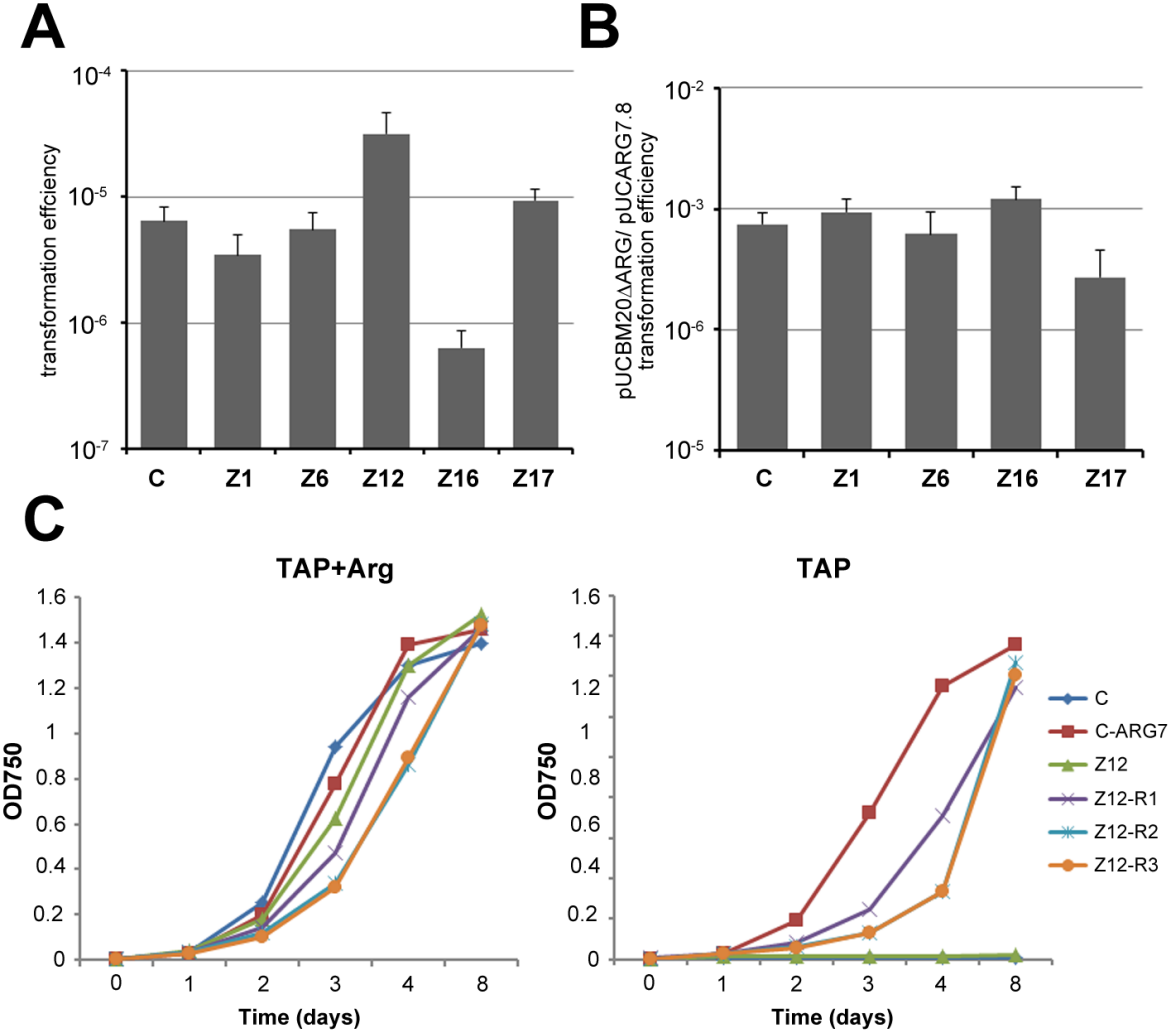
Transformation efficiency of *C. reinhardtii* mutants. (A) Transformation efficiency calculated as the frequency of Arg-prototrophic transformants per a total number of transformed cells normalized to 1 pmol of pUCARG7.8 construct used for transformation. (B) Efficiency of homology-driven integration estimated as a ratio between the transformation efficiencies obtained with the pUCBM20ΔARG and pUCARG7.8 constructs. (A, B) C = *cw15-302 arg2*; standard errors of three independent experiments are indicated (N = 3). (C) Growth curves of the Z12 strain and selected Z12 Arg-prototrophic revertants in TAP liquid media with or without Arg. *cw15-302 arg2* cells complemented with the *ARG7* construct were used as an Arg-prototrophic control (C-ARG7).

Insertion of exogenous DNA into a genome during transformation is governed by processes involved in DSB repair. To examine whether the efficiency of DNA integration via NHEJ or homology driven repair is altered in the isolated strains, we took advantage of the missense mutation *arg7-8* in the *ARG7* gene that encodes argininosuccinate lyase (ASL) and confers arginine auxotrophy of the *cw15-302 arg2* strain [21]. We transformed the mutant strains with plasmids carrying either the entire *ARG7* gene (pUCARG7.8), or a truncated version that lacks the 5′ region encoding the promoter and N-terminal portion of ASL (pUCBM20ΔARG) [21], [28]. Since the transformants were selected for complementation of ARG7 function by their ability to grow on plates without arginine, prototrophy of the pUCBM20ΔARG transformants was anticipated to be caused by homology driven repair that corrected the *arg7-8* mutation [21]. While transformation efficiency with the pUCARG7.8 construct was unaffected in Z1, Z6 and Z17 mutants as compared to the *cw15-302 arg2* strain, a 10-fold decrease was detected in the Z16 background suggesting deficiency in NHEJ (P = 0.063, two-tailed Student’s t test; Figure 2A). Interestingly, occurrence of Arg prototrophic clones increased by one order of magnitude in Z12. Homology driven integration in *C. reinhardtii* has been reported to be 1.5–4 orders of magnitude lower than random integration and depends on the transformation method and constitution of transformed DNA [20], [30], [31], [32]. In our conditions, the frequency of pUCBM20ΔARG transformants in the *cw15-302 arg2* strain was approximately 1000-times lower than of pUCARG7.8. Similar ratios were detected in the Z1, Z6, and Z16 strains while a slight decline in the ratio was detected in Z17, indicating a deficiency in HR (P = 0.088, two-tailed Student’s t test; Figure 2B).

The high frequency of Arg prototrophs recovered from the Z12 background was unusual and warranted further verifications. We found that prototrophic colonies appeared in a transformation-independent manner and hence, represented spontaneous phenotypic reversions. These reversions are specific to the Z12 strain and were never observed in the parental or other mutant strains. The Arg prototrophic phenotype of Z12 revertant clones was stable, but they appeared to grow slower in liquid media without arginine than *ARG7* complemented *cw15-302 arg2* cells (Figure 2C). Sequencing of the *ARG7* region in several Z12 revertants excluded the possibility that the phenotypic reversions were caused by spontaneous repair of the *arg7-8* mutation (data not shown). Thus, we conclude that Z12 cells are predisposed to activate an adaptive mechanism enabling them to overcome *arg7-8* deficiency mediated arginine auxotrophy.

**Figure 3:**
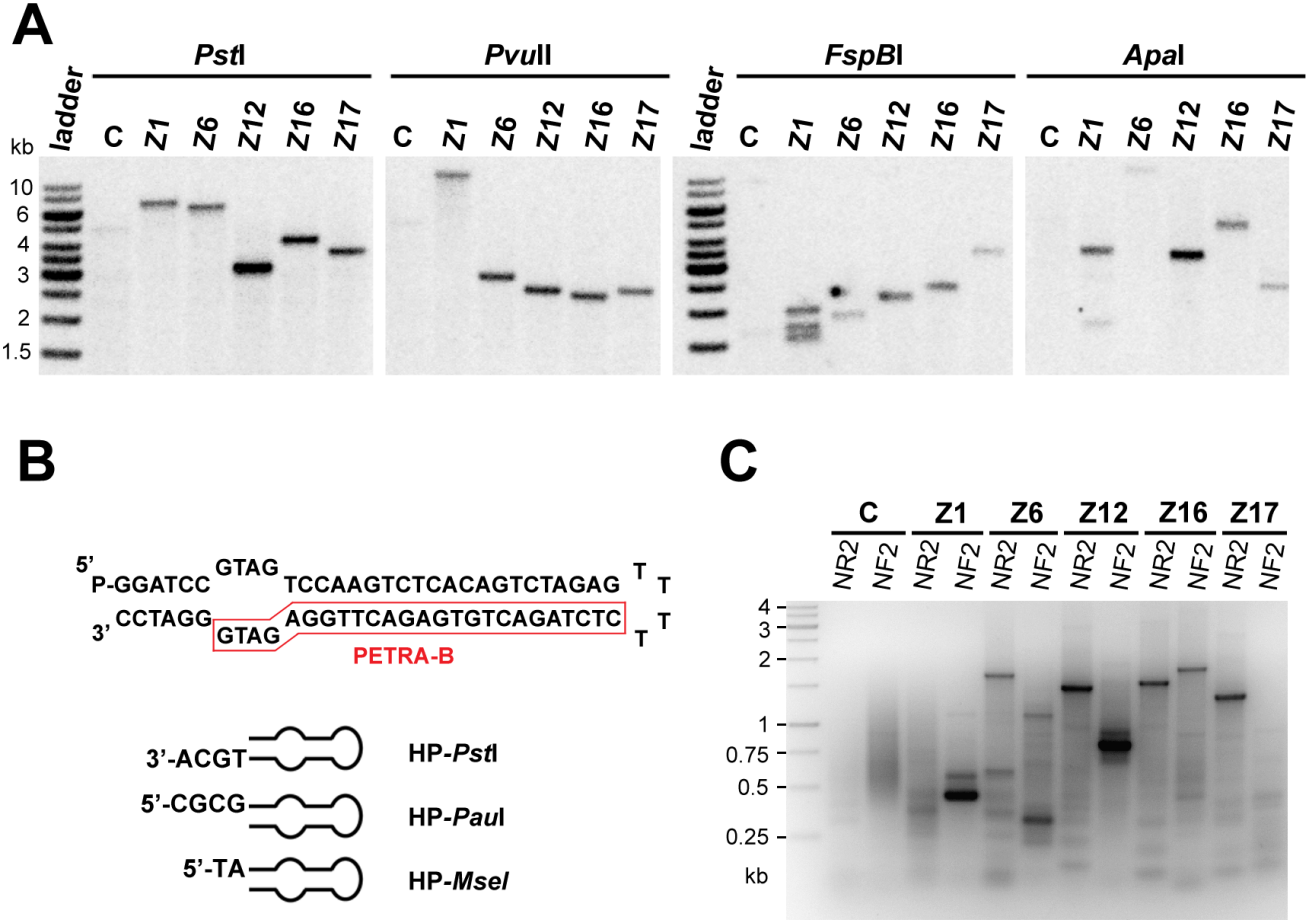
Molecular characterization of *aph7*″ insertions. (A) Southern analysis of *aph7*″ insertions in individual mutants. The restriction enzymes used for each experiment are indicated; positions of the restriction sites within the *aph7*″ construct probe used for hybridization are depicted in Figure 1A. (B) Structure of the hairpin adaptor with a blunt end; examples of hairpins with ends compatible with selected restriction enzymes are indicated. (C) PCR products, separated in agarose gels and stained with ethidium bromide, that were generated after two rounds of nested PCR from a genomic library produced by *Pst*I digest.

Next we sought to identify the loci disrupted by insertional mutagenesis. First, we determined the number of independent insertions in the genomes of the mutant strains by Southern analysis with a probe specific for the insertional cassette (Figure 1A, 3A). Restriction analysis with enzymes that digest outside of the construct (*Pst*I, *Pvu*II) revealed the presence of a single insertion in all mutants (Figure 3A). Multiple bands that appeared in the Z1 mutant after digestion with enzymes that cleave within the *aph7*″ gene (*Fsp*BI, *Apa*I; Figure 1A, 3A) were suggestive of three copies of the insert in a single site. Other mutants appeared to harbour only a single copy of *aph7*″ in their genome.

**Figure 4:**
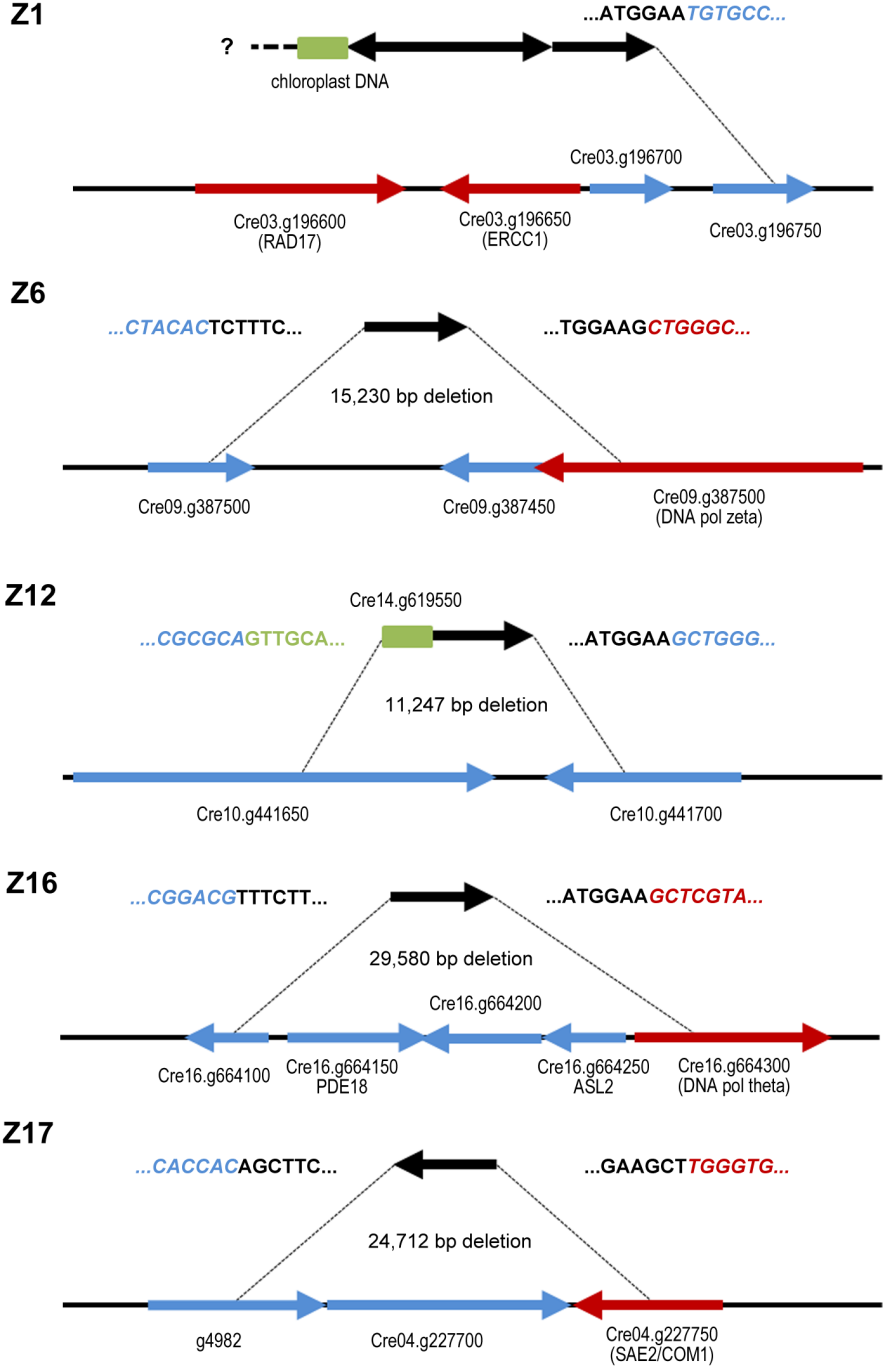
Structure of insertion sites. Black arrows represent the aph7″ insert, red arrows indicate homologues to known DNA repair genes, blue arrows represent genes unrelated to DNA repair, and green boxes depict DNA fragments that were co-transformed to the insertion sites from ectopic locations. Sequences at the insert borders are indicated.

To localize the insertion sites, we developed a PCR-based strategy utilizing ligation of DNA hairpin adaptors to digested genomic fragments (Figure 3B). We have originally designed these adaptors to specifically amplify blunt-ended telomeres in *A. thaliana* [27]. The end of the hairpin can be designed to contain complementarity to DNA ends generated by any restriction enzyme (Figure 3B). The adaptor further harbours a short stretch of non-complementary sequence forming a bubble near the open end of the hairpin. This bubble corresponds to the sequence of a PCR primer (PETRA-B; Figure 3B) that does not amplify ligated adaptor dimers, but can only anneal to DNA strands arising from the extension of a primer specific to a gene of interest. To amplify sequences flanking the insertion site, ligation of the adaptor to digested genomic DNA was followed by two rounds of nested PCR with the PETRA-B and insert specific primers (Figures 1A and 3C). Several adaptor-ligated libraries generated by different restriction enzymes were usually tested for each mutant. The most suitable restriction enzymes for this purpose were *Pst*I, *Pau*I, *Pvu*II, *Nsb*I and *Mse*I. PCR products were sequenced and aligned to the scaffold of the *C. reinhardtii* genome. Using this approach we identified both flanking sites in Z6, Z12, Z16 and Z17 mutants. Insertions in these strains were accompanied by large deletions ranging from 11 to 30 kb that encompassed two to five genes (Figure 4). Deletions in Z6, Z16 and Z17 disrupted genes coding for proteins homologous to the known DNA repair factors DNA Pol zeta, DNA Pol theta and SAE2/COM1 nuclease, respectively. An 11 kb deletion in Z12 affected two proteins of unknown function, Cre10.g441650 and Cre10.g441700, which show no extensive sequence homology in protein databases and appear to be specific to *Chlamydomonas* and closely related genomes, e.g. *Volvox carteri*. Only one flanking site with homology to the nuclear genome was retrieved in Z1 mutants. The other insertion border contained chloroplast DNA (Figure 4). The Z1 insertion was localized to the vicinity of genes coding for the ERCC1 endonuclease and the RAD17 DNA damage checkpoint protein. PCR analyses demonstrated that both genes are absent in the Z1 mutant, indicating that a large deletion occurred at the insertion site in this strain. We attempted to complement the zeocin sensitivity phenotypes by transforming the mutant strains with BAC clones spanning the deleted regions. However, despite extensive efforts, these experiments failed due to technical difficulties transforming *C. reinhardtii* with large DNA fragments. Therefore, we could not ascertain that the DNA damage sensitivity of mutant strains was indeed caused by disruption of genes at the insertion loci.

## Discussion

The use of forward genetic screens for discovery of genes involved DNA repair and DNA damage response has been limited in plants; most plant DDR/DNA repair proteins were identified based on sequence homology with their yeast and mammalian counterparts. The major caveat of this homology driven approach is its inability to identify genes and processes that may have evolved specifically in the plant lineage to deal with genomic insults. The importance of unbiased approaches is documented by a screen in *A. thaliana* that revealed novel genes linking DDR with epigenetic regulation and stress signalling pathways [13], [14]. Similarly, SOG1, a transcription factor governing DDR is a plant specific protein that was discovered in a screen to identify suppressors of the radiosensitivity of a nucleotide excision repair mutant [33].

Here we exploited the unicellular green algae *C. reinhardtii* in a forward genetic screen for the discovery of DDR/DNA repair genes. By surveying a mutagenized population of *C. reinhardtii*, we have identified five transformants that exhibited stable sensitivity to the radiomimetic drug zeocin and, with exception of the mutant Z16, to at least one other genotoxic treatment. To identify the loci disrupted in these mutants, we have developed a novel PCR-mediated strategy based on ligation of hairpin adapters to genomic fragments generated by restriction endonucleases cutting within G/C-rich sequences. Inverse PCR and TAIL-PCR are the usual methods of choice for cloning sequences flanking insertion sites, but the high GC content of the *C. reinhardtii* genome renders the success of these techniques highly variable [34], [35]. With the hairpin-ligation strategy we were able to readily obtain sequences flanking both insertion sites for all mutants; in fact, in some mutants we obtained these sequences several times independently using different restriction enzymes (data not shown). The forward genetic screen in *C. reinhardtii* was extremely time efficient in comparison to other plant models as the entire procedure from making the mutant library to the identification of disrupted loci took about 9 months. Validation of the DNA-damage sensitivity proved to be the most critical and time consuming step as a large proportion of isolated mutants lost the phenotype after 3 to 6 months of subculturing. Lack of tools for efficient validation of genes identified in the screen was the major hurdle we faced in this study. Insertional mutagenesis led to deletions spanning several genes. As *C. reinhardtii* is not amenable to gene targeting, genetic complementation appears to be a method of choice for confirming association between scored phenotypes and disrupted loci. However, also this approach can be in *C. reinhardtii* troublesome. *C. reinhardtii* genes can span tens of kbs which in combination with very high CG content impedes classical cloning and PCR based approaches for making complementing constructs. For these reasons, we were unable to complement mutant phenotypes and formally confirm identity of causative genes. We anticipate that advances in genome-editing technologies utilizing TALENs or CRISPR/Cas9 will eventually solve this issue and facilitate gene discovery in *C. reinhardtii*. Nevertheless, the fact that four out of five insertion-tagged loci contained disruption of known DNA repair genes validates suitability of the experimental approach in discovery of genes involved in plant DDR.

In the case of the Z1 insertion we were able to map only one border to the nuclear genome, while the other flanking sequence consisted of a chloroplast DNA fragment. The insertion event led to deletion of genes homologous to *ERCC1* and *RAD17*. ERCC1 forms, together with XPF, a structure specific nuclease discovered for its role in nucleotide excision repair [36]. It is also involved in processing of DNA interstrand crosslink lesions and homologous recombination intermediates. ERCC1 deficiency in *A. thaliana* has been reported to cause sensitivity to MMC, MMS and UV and gamma irradiation [15]. RAD17 is a conserved replication checkpoint protein important for genome stability and its inactivation in *A. thaliana* renders plants sensitive to bleocin and MMC [37]. Thus, the sensitivity of *C. reinhardtii* Z1 mutants to a broad range of genotoxic treatments is likely caused by the combined absence of these DNA repair proteins.

Characterization of insertion sites in the Z6 and Z16 mutants revealed deletions disrupting genes coding for translesion synthesis DNA polymerases zeta and theta, respectively. DNA Pol zeta can bypass a range of DNA lesions including adducts induced by cisplatin, UV and apurinic/apyrimidinic sites [38] and there is evidence suggesting its function in interstrand crosslink repair and HR [39], [40]. This is consistent with the relatively mild, but broad sensitivity of the Z6 strain to genotoxic insults that include zeocin, UV and MMC; a similar spectrum of sensitivity was also observed in the *A. thaliana* DNA pol zeta mutant [41]. In contrast, the Z16 strain carrying a deletion in DNA pol theta (also known as POLQ) exhibits strong sensitivity to zeocin, but not to the other treatments. Interestingly, *A. thaliana* pol theta deficient plants display developmental defects, constitutive DNA damage response and sensitivity to MMC and MMS [42]. Thus, this polymerase may have acquired more fundamental role in DNA repair in higher plants. Mouse and human cell lines impaired in DNA pol theta are sensitive to bleomycin and ionizing radiation, implicating its function in some aspects of DSB repair [43], [44]. Studies in *Drosophila melanogaster* indicated that DNA Pol theta contributes to DSB repair by facilitating microhomology-mediated DNA end-joining [45], which is in accordance with our observation of a 10-fold decrease in the transformation efficiency of the Z16 mutant.

The Z17 strain harbours a deletion that disrupts SAE2/COM1/CtIP, a protein that co-operates with the MRE11-RAD50-NBS1/Xrs2 nuclease to initiate resection of DSB during HR [46], [47]. This complex also plays a central role in the repair of stalled or collapsed replication forks, and mutants described in a number of organisms are sensitive to a wide range of DNA damaging agents including MMC, ionizing radiation, hydroxyurea, bleomycin or MMS [48], [49], [50]. The conserved HR function of SAE2/COM1 in *C. reinhardtii* is also indicated by decreased efficiency of homology-driven integration of the pUCBM20ΔARG construct into Z17 genome. In higher plants, COM1 appears to participate in interstrand crosslink repair as *A. thaliana com1* mutant displayed sensitivity to MMC [51].

Identification of several well characterized DNA repair factors at the insertion sites in Z1, Z6, Z16 and Z17 strains demonstrated the ability of the screen to uncover genes involved in DDR and genome integrity. Nevertheless, the ultimate goal of forward genetic screens is to identify unknown genes or unanticipated links to the processes under study. Two previous studies reported on the identification of novel genes involved in DNA repair in *C. reinhardtii* by combining insertional mutagenesis with a screen for sensitivity to genotoxic treatments [52], [53]. The disruption in the Z12 mutant also affects two genes encoding proteins of unknown function that appear to be specific to *C. reinhardtii* and closely related species. The Z12 mutant exhibits the strongest sensitivity to HU and MMS among all the mutants recovered in our screen, suggesting the impairment of processes associated with DNA replication. Interestingly, we observed that in addition to DNA damage sensitivity, Z12 cells are able to overcome arginine auxotrophy caused by the *arg7-8* mutation in the *ARG7* gene. Mutations in the *ARG7* have been used for decades as auxotrophic markers in *C. reinhardtii* transformation experiments for their stability and rare reversions [54], [55]. However, the spontaneous phenotypic reversions to arginine prototrophy occur in Z12 at an extremely high frequency and are not accompanied by correction of the causative mutation in the *ARG7* gene. The adaptive mechanism that is activated in Z12 revertants is unknown. It is also unclear whether the propensity of Z12 cells to switch to Arg prototrophy is linked to the DNA repair deficiency phenotype and whether it associates with either of the disrupted genes. The high rate of the phenotypic reversion, along with its stability, evokes parallels with de-repression of an epigenetically suppressed mechanism. Spontaneous changes in epigenetic states can be stably inherited and occur at much higher frequencies than genetic mutations. Furthermore, malfunction of numerous DNA replication and repair factors has been linked to de-repression of epigenetically silent loci, and, *vice versa*, deficiency in chromatin regulators may lead to impaired DNA repair [13], [56], [57], [58]. Thus, we speculate that the phenotypic switch to arginine prototrophy in Z12 cells is conditioned by a mutation in a gene that is required for both genome integrity and epigenetic inheritance. While further in depth mechanistic studies are required to decipher this phenomenon, the example of the Z12 mutant illustrates the power of forward genetics in *C. reinhardtii* in uncovering novel genes and processes involved in genome maintenance.
